# Economic Feasibility of Floating Offshore Wind Farms Considering Near Future Wind Resources: Case Study of Iberian Coast and Bay of Biscay

**DOI:** 10.3390/ijerph18052553

**Published:** 2021-03-04

**Authors:** Laura Castro-Santos, Maite deCastro, Xurxo Costoya, Almudena Filgueira-Vizoso, Isabel Lamas-Galdo, Americo Ribeiro, João M. Dias, Moncho Gómez-Gesteira

**Affiliations:** 1 Departamento de Enxeñaría Naval e Industrial, Escola Politécnica Superior, Universidade da Coruña, Esteiro, 15471 Ferrol, Spain; 2 Environmental Physics Laboratory (EphysLab), Centro de Investigacións Mariñas (CIM)-UVIGO, Universidade de Vigo, Edificio Campus da Auga, 32004 Ourense, Spain; mdecastro@uvigo.es (M.d.); jorge.costoya.noguerol@usc.es (X.C.); mggesteira@uvigo.es (M.G.-G.); 3 Group of Nonlinear Physics, Department of Particle Physics, CRETUS Institute, University of Santiago de Compostela, 15705 Santiago de Compostela, Spain; 4 Departamento de Química, Escola Politécnica Superior, Universidade da Coruña, Esteiro, 15471 Ferrol, Spain; almudena.filgueira.vizoso@udc.es; 5 Departamento de Ciencias da Navegación e Enxeñaría Mariña, Escola Politécnica Superior, Universidade da Coruña, Esteiro, 15471 Ferrol, Spain; isabel.lamas.galdo@udc.es; 6 Centro de Estudos do Ambiente e do Mar (CESAM), Physics Department, University of Aveiro, 3810-193 Aveiro, Portugal; americosribeiro@ua.pt (A.R.); joao.dias@ua.pt (J.M.D.)

**Keywords:** floating offshore wind farms, wind power density, CORDEX future projections, *LCOE*, *NPV*, *IRR*

## Abstract

Wind energy resources are subject to changes in climate, so the use of wind energy density projections in the near future is essential to determine the viability and profitability of wind farms at particular locations. Thus, a step forward in determining the economic assessment of floating offshore wind farms was taken by considering current and near-future wind energy resources in assessing the main parameters that determine the economic viability (net present value, internal rate of return, and levelized cost of energy) of wind farms. This study was carried out along the Atlantic coast from Brest to Cape St. Vincent. Results show that the future reduction in wind energy density (2%–6%) mainly affects the net present value (*NPV*) of the farm and has little influence on the levelized cost of energy (*LCOE*). This study provides a good estimate of the economic viability of OWFs (Offshore Wind Farms) by taking into account how wind resources can vary due to climate change over the lifetime of the farm.

## 1. Introduction

Renewable energies are an inexhaustible source of energy available on our planet within the reach of human beings. In the 10th and 11th centuries, the first technological advances took place in Europe, which placed hydraulic energy at the center of economic life, through water mills. In areas where water was scarce, many towns resorted to the use of windmills (the first windmill was built in Yorkshire (England) in 1185 [1]).

However, renewable energy only began to be explored on a regular basis after the 1980s due to the weakening of the ozone layer and the awareness of the damage to the planet caused by greenhouse gases. Energy sources such as the sun, wind, or water have held an important place in the world’s energy systems and have continued to grow as technological improvements make their use more profitable and efficient.

As in Europe, in Spain, the evolution of renewable energies began in the 1990s, but the increase in installed power was noticed only at the beginning of the 21st century, growing from 643 MW in the 1990s to 110,000 MW installed in 2020 [2]. 

In 2000, 70% of all wind energy installed in the world was in Europe. With the evolution of onshore wind energy and the possibilities that it offered, the search for new locations at sea began, but these lacked new technologies for the installation of wind farms. In an almost symbolic way, the Elkraft company (predecessor of the current DONG Energy) installed its first wind farm in Denmark in 1991. The “Vindeby Offshore Wind Farm” had 11 wind turbines from the manufacturer Bonus Energy with a power of 450 kW each and with a total farm power of 4.95 MW. Following in the footsteps of Denmark, OWFs (Offshore Wind Farms) began to be installed around the world. Currently, the continent of Europe has more offshore wind power installed (3.6 GW) than any other. This trend is expected to continue in the future, and it is forecasted that in 2050 between 230 and 450 GW of offshore wind energy will be installed to comply with the Green Pact [3,4,5].

Regarding wind, there are numerous studies about different new issues: the effect of wake effect in offshore farms [6], the layout of the offshore farms [7], the types of offshore substructures [8], the economics of offshore wind, repowering wind farms [9], how to combine wind and wave energy [10], etc.

Previous studies on marine energy production in the Iberian Peninsula covering the period 2000–2013 [11,12,13,14] show the highest wind power energy in the northwestern corner of the Iberian Peninsula, reaching annual mean values in the range of 300–350 W m^–2^, based on wind data at the height of 10 m, and 500 Wm^–2^ from wind data at the height of 120 m. Furthermore, a similar wind power flux was observed in the Strait of Gibraltar from the wind data at the height of 120 m, and wind power values of 400–450 W m^–2^ were recorded near Cape Roca and Cape St. Vincent. The wind data used to calculate the wind power flux were obtained from different sources, such as QuikScat, ASCAT/OSCAT scatterometers, reanalysis databases (ERA-Interim, NCEP-CFSR, NCEP-R2, NCEP-FNL, NCEP-GFS, NASA-MERRA), NCDC and IFREMER blended wind fields, the Cross-Calibrated Multiplatform (CCMP) ocean wind vectors, and a high-resolution Weather Research and Forecast (WRF) model of offshore wind data. Similar studies [15,16,17,18] carried out using wind measurements collected from marine buoys and several WRF model simulations reported wind energy densities between 300 and 700 W m^–2^ at these locations (at 10 m above sea surface level (asl)), with the highest values found around Cape Finisterre on the northwestern corner of the Iberian Peninsula. Other authors [19] also concluded that the Atlantic coast of the Iberian Peninsula is a suitable location for offshore wind energy exploitation with mean annual energy density reaching up to 950, 450, and 400 W m^−2^ in the northern, central, and southern regions, respectively. These results were obtained using WRF model simulations at 9 and 3 km spatial resolution and 6-hourly output at 10 m asl for a 10-year wind hindcast period (2004–2013).

The impact of climate change on wind energy production was also previously analyzed on the Atlantic coast of the Iberian Peninsula. Global analysis of the wind energy output in the whole of Europe performed using a multimodel ensemble of EURO-CORDEX regional climate models (RCMs) predicts that by the end of the 21st century, there will be an overall decrease in western Iberia, with the exception of the northwestern corner where null changes were detected [20,21,22]. A similar study done with a more regional focus [23] projected a 2.5% to 10% yearly reduction in wind speed and wind power over the entire western coast, except for the northwestern area where no changes were expected due to the wind increase projected in summer. Additionally, [24] also found that the wind power density (WPD) decrease projected by the end of the 21st century is not homogeneous throughout the year. In particular, a WPD increase is projected for summer in the entire area, and a WPD decrease is projected for spring and, especially, for autumn. Regarding winter, a WPD increase is projected in the northern area, and a reduction is expected for the rest of the region. Previous regional studies [22,25] associated the weakening of the westerly wind with the strength of the Azores High and its northeastward displacement over the 21st century. A complete review of offshore wind energy resources in Europe, and in the Iberian Peninsula in particular, under present and future climate can be found in [26].

Despite the huge amount of studies dealing with the wind resource and the impact of climate change on wind resource in the area under study, the impact of climate change on the economic feasibility of floating OWFs has never been considered before. In fact, previous studies on the profitability of OWFs based their results on the wind resource data of recent years [27,28,29,30,31]. However, it is important to note that feasibility studies are being developed to predict how the farm will perform in the near future. Therefore, the wind energy considered for the economic parameters should be that calculated for the future.

The aim of the present study is to analyze the economic viability of floating OWFs along the Atlantic coast from Brest to Cape St. Vicente both now and in the near future. This study represents a step forward in determining the profitability of OWFs in the near future by taking into account future wind projections in the context of climate change. For this purpose, a multimodel ensemble of EURO-CORDEX RCM simulations was used to determine the WPD for the 2001–2020 (historical) and 2021–2040 (near future) periods. Then, several cases were analyzed considering the past and future energy data, constant and variable, in order to assess the differences in terms of economic profitability. For each of the cases analyzed, the economic feasibility parameters of an OWF were calculated, namely the internal rate of return (*IRR*), the net present value (*NPV*), and the levelized cost of energy (*LCOE*).

## 2. Materials and Methods 

Current (2001–2020) and near future (2021–2040) wind patterns retrieved from the EURO-CORDEX project (http://www.cordex.org/ (accessed on 20 June 2020) were used to analyze the economic profitability of floating OWFs. To achieve this goal, a multimodel approach was considered based on the output of seven different RCMs whose accuracy in reproducing real wind conditions for the area under study was assessed in previous research. Wind data were used to calculate the main parameters that determine the economic viability of wind farms, namely net present value, internal rate of return, and levelized cost of energy. Four methods were considered taking into account the approximation of calculating the energy generated by the floating OWF:

- Method 1: Average of future prediction for all years (energy constant with future data);
- Method 2: Future prediction for each year (energy variable with future data);
- Method 3: Average of past prediction for all years (energy constant with past data);
- Method 4: Past data for each year (energy variable with past data).

### 2.1. Data

Daily wind speed data at 10 m asl were retrieved from seven simulations carried out within the framework of the EURO-CORDEX project with a spatial resolution of 0.11° × 0.11°. A total of four RCMs driven by four GCMs were considered both for the historical period (2001–2020) and the near future (2021–2040) under the RCP8.5 greenhouse gas (GHG) emission scenario (Table 1). The RCP8.5 scenario represents a GHG emission that will lead to 8.5 W m^−2^ of radiative forcing by the end of the 21st century and will continue to increase after 2100. This GHG scenario is considered the most realistic today due to the lack of effective actions to reduce GHG emissions worldwide. Finally, a multimodel ensemble approach was considered because previous studies [32,33] have shown that it minimizes errors and uncertainties associated with each individual model.

The ability of this multimodel ensemble to reproduce wind speed at 10 m asl in the study area was previously demonstrated by comparing numerical wind speed series with in situ data from buoys using different statistical metrics (percentage of error, overlap percentage, root-mean-square error, and percentage of difference in median wind speed series) [18,24,34]. In particular, according to F. Santos et al. (2018), the percentage of error between the mean wind projected for each RCM and the mean wind at each buoy is around 10% for the whole Iberian Peninsula and around 8% for the buoys located in the area under study. Additionally, the overlap percentage between the distributions of winds measured by buoys and estimated by RCMs shows values higher than 83% ± 3% and higher than 85% ± 3% for the area under study.

### 2.2. Methodology

The *WPD* (W m^−2^) of a wind turbine can be calculated according to [35] by Equation (1):

(1) $$WPD=\frac{1}{2}\rho_{a}W_{H}{}^{3}$$

where $W_{H}$ is the wind speed (m s^−1^) at the turbine hub height (120 m) and *ρ_a_* is the air density (1.225 kg m^−3^ at 15 °C and 1000 hPa). Although this expression is widely used to calculate wind power densities, it is necessary to take into account that it underestimates the wind energy since WPD depends on the Weibull distribution [36] and, therefore, more power exists above the mean wind speed than that predicted by averaging.

Wind data from CORDEX project were extrapolated from 10 m to the hub height of the offshore turbine (120 m) considering a logarithmic wind profile that assumes a neutrally stratified atmosphere [37], Equation (2), applied in previous studies [38,39]:

(2) $$W_{H}=W_{ns}\frac{\ln\left(\frac{H}{z_{0}}\right)}{\ln\left(\frac{H_{ns}}{z_{0}}\right)}$$

where *H* is the hub height of the turbine, *H_ns_* is the height of near-surface winds (10 m), *W_H_* is the wind speed at the hub height, *W_ns_* is the near-surface wind speed, and *z_0_* is the roughness length. A value of 0.001 m was considered for *z_0_* assuming an open calm sea [40]. This option was selected because the CORDEX project does not contain all necessary variables for calculating atmospheric stability at each time and pixel to develop the Monin–Obukhov theory [41] or similar.

WPD, expressed in watts per square meter, represents the energy available to be converted by a wind turbine at a specific location. This variable is independent of the specific properties of wind turbines, allowing for comparison of the wind resource at different locations. 

One of the factors deterring many countries from choosing to exploit offshore wind energy in their energy matrix is its profitability. There are many factors that influence profitability, and one of the most important is the location of the farms. In this context, geographic information system (GIS) software is a conceptual framework that provides the ability to capture and analyze spatial and geographic data. GIS software has proven to be very useful in establishing optimal locations for wind farms, as supported by numerous previous studies [42,43,44,45,46].

Here, the economic feasibility of the farm is determined by considering the typical economic parameters for investment projects in the energy industry: the net present value (*NPV*), the internal rate of return (*IRR*), and the levelized cost of energy (*LCOE*).

*NPV* is the “net value of the cash flows of the floating OWF considering its discount from the beginning of the investment” [47,48]. It is a function of the cash flow of the project on year *n* ($CF_{n}$), the discount rate considered (r), and the initial investment of the project ($I_{0}$), as represented in Equation (3).

(3) $$NPV=-I_{0}+\overset{N_{farm}}{\underset{n=1}{\sum }}\frac{CF_{n}}{{\left(1+r\right)}^{n}}$$

*IRR* is the discount rate when the *NPV* is equal to zero (see Equation (4)) [47,48].

(4) $$0=-I_{0}+\overset{N_{farm}}{\underset{n=1}{\sum }}\frac{CF_{n}}{{\left(1+IRR\right)}^{n}}$$

Moreover, *LCOE* takes into account the total costs of the farm ($LCS_{FOWF_{n}}$) in €, the capital cost of the project (r), and the energy produced by the farm ($E_{n}$) in MWh/year [47], as shown in Equation (5).

(5) $$LCOE=\frac{\sum_{n=0}^{N_{farm}}\frac{LCS_{FOWF_{n}}}{{\left(1+r\right)}^{n}}}{\sum_{n=0}^{N_{farm}}\frac{E_{n}}{{\left(1+r\right)}^{n}}}$$

**Figure 1:**
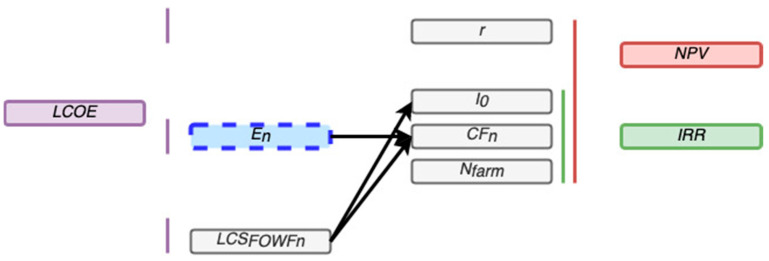
Relation between the economic feasibility parameters.

The cash flow of the project ($CF_{n}$) in the year *n* depends on the energy produced by the floating OWF in this *n* year. Therefore, *NPV*, *IRR*, and *LCOE* are dependent on the energy produced (see Figure 1). 

### 2.3. Case Study

**Figure 2:**
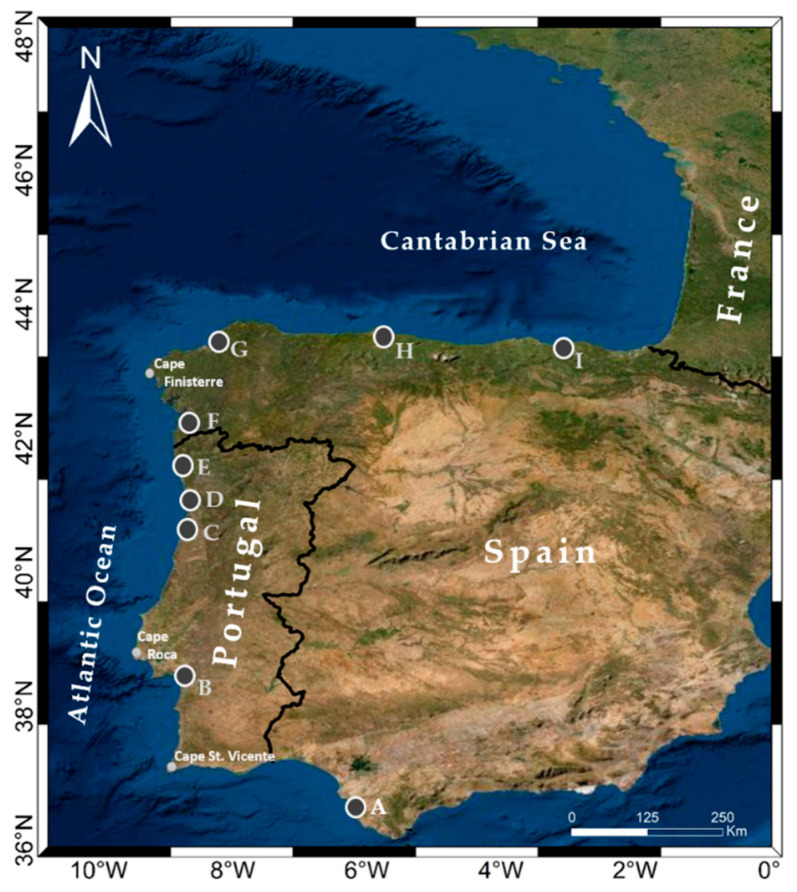
Area under study. White circles with letters are the locations of the main harbors supporting ocean renewable energies logistics. **A** Puerto Real Cádiz (36.51° N, −6.24° W), **B** Setubal (38.52° N, −8.89° W), **C** Aveiro (40.64° N, −8.75° W), **D** Leixoes (41.18° N, −8.70° W), **E** Viana do Castelo (41.69° N, −8.84° W), **F** Vigo (42.23° N, −8.74° W), **G** Ferrol (43.48° N, −8.25° W), **H** Gijón (43.55° N, −5.70° W), and **I** Sestao (43.35° N, −3.05° W). Source: Own elaboration.

The area of study to analyze the feasibility of floating OWFs covers the west coast of the Iberian Peninsula (Spain and Portugal) and the Bay of Biscay (see Figure 2). 

The calculations were completed considering the WindFloat floating offshore wind platform of 5 MW designed by PrinciplePower [49,50].

The main characteristics of the farm considered are shown in Table 2.

## 3. Results

### 3.1. Offshore Wind Resource

**Figure 3:**
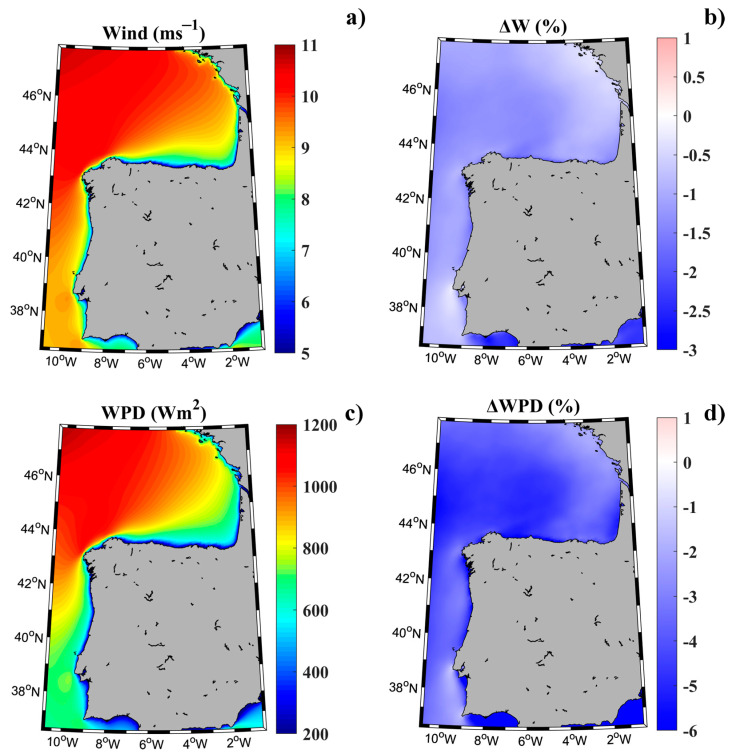
(**a**) Future wind speed (ms^−1^) at the height of the hub (120 m asl) projected for the 2021–2040 period, (**b**) future increase of wind speed (in percentage) with respect to the historical period (2001–2020) (**c**) future wind power density (W m^−2^) projected for the 2021–2040 period, and (**d**) future increase of wind power density (in percentage) with respect to the historical period (2001–2020). Future projections were calculated under the RCP8.5 gas emission scenario. Source: Own elaboration.

Wind resource (m s^−1^) and wind power density (WPD W m^−2^) projected under the RCP8.5 greenhouse gas emission scenario for the near future (2021–2040) are shown in Figure 3a,c. Furthermore, the projected increases in wind and WPD (in percentage) with respect to the historical period (2001–2020) are shown in Figure 3b,d.

The future wind resource will reach values higher than 8.5 m s^−1^ for most of the region, except in a fringe close to the coast where the wind resource diminishes to values between 5 and 8 m s^−1^. This fringe is wider along the Cantabrian Sea than in the rest of the region. The wind resource will decrease by between 0.5% and 1.5% for practically the whole northwest coast of the Iberian Peninsula in the near future. Only in some specific areas, such as in front of Cape Finisterre and between Cape Roca and Cape San Vicente, are there no significant changes in the wind resource for the near future. The area of the northwestern corner is coincident with the region where an increase (~3%) in WPD is projected by the end of the century in [24].

The WPD will reach values between 300 and 700 W m^−2^ near the coast for the whole region, except between Cape Finisterre and Cape Ortegal, where it will reach values between 900 and 1000 W m^−2^. The WPD will only achieve values close to 1100 W m^−2^ at offshore locations, in the northwest corner of the study area. The future decrease in WPD will be greater (between 2% and 6%) than that observed for the wind resource. This was to be expected, since, following Equation (3), any small variation in wind speed has a large influence on the potential, as it is proportional to the cube’s wind speed. The smallest decrease in WPD was observed off Cape Finisterre and between Cape Roca and Cape San Vicente, as was previously observed for the wind resource.

The WPD reduction detected for the near future in the study area agrees with results obtained in [24] by means of corrected projections of a CORDEX RCM ensemble for the near future (2025–2040). The WPD decrease obtained in the present study also agrees with previous research on the impact of climate change on future European wind energy resources. More specifically, a weak reduction (2%–6%) in the future wind power potential was detected over most of northern Europe during the next 30–40 years by downscaling coarse results from coupled GCMs under the A1B scenario from 2020 to 2049 [51]. More recently, ensemble mean projections revealed a decrease of mean annual WPD for most of Europe in future decades, while increases were found for the Baltic and Aegean Seas [20,38].

### 3.2. Economic Results

**Figure 4:**
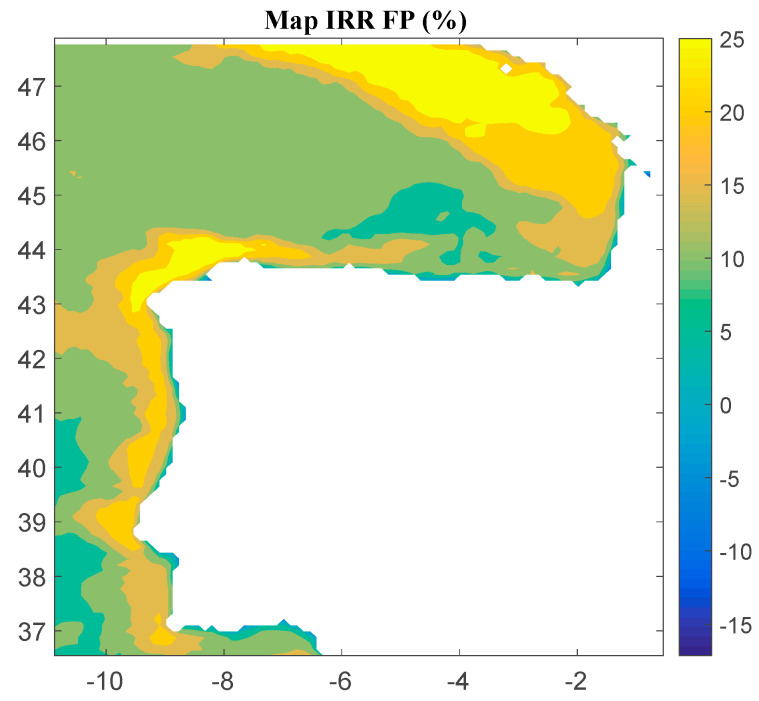
*IRR* (in percentage) for the floating OWF selected using Method 1. Source: Own elaboration.

**Figure 5:**
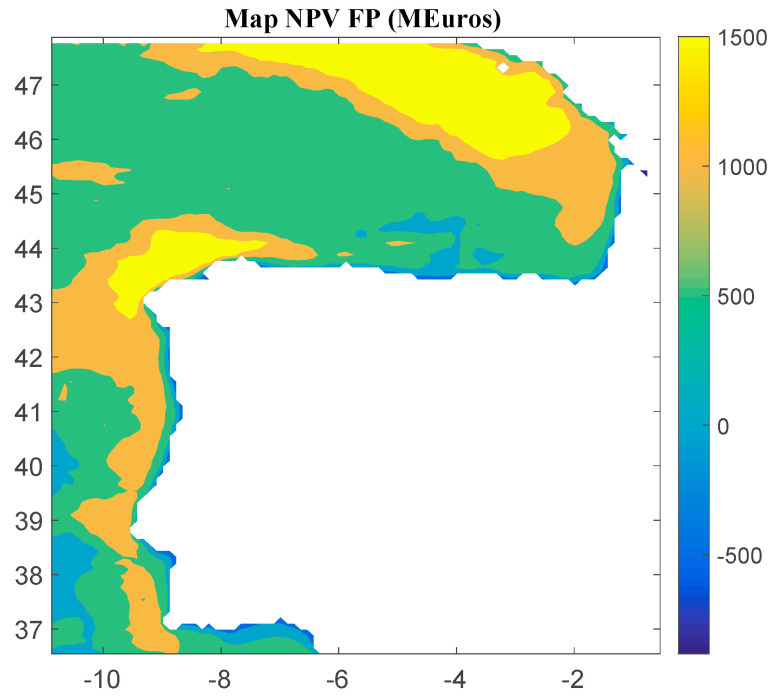
*NPV* (in M€) for the floating OWF selected using Method 1. Source: Own elaboration.

**Figure 6:**
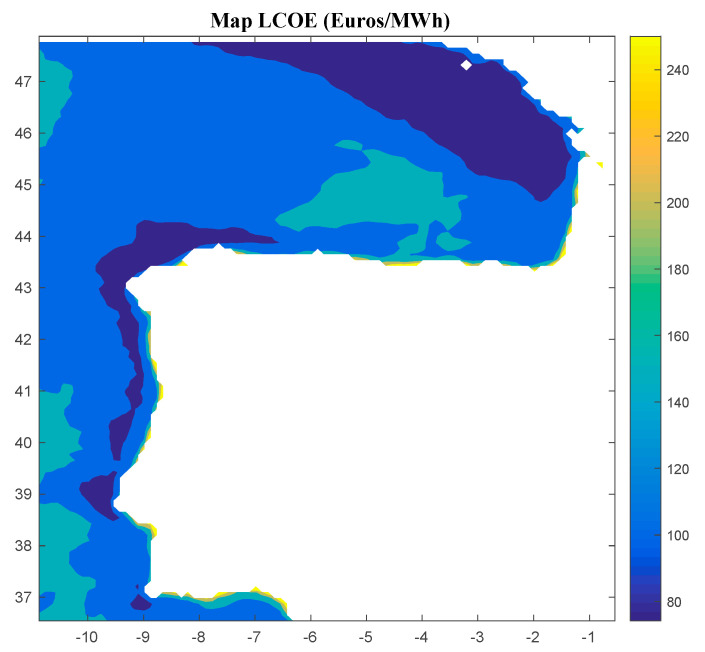
*LCOE* (in €/MWh) for the floating OWF selected using Method 1. Source: Own elaboration.

Considering Method 1, the maps for *IRR*, *NPV*, and *LCOE* are shown in Figure 4, Figure 5 and Figure 6, respectively. The *IRR* ranges from −17% to 29%, and *NPV* has values ranging from −882 to 1948 million of euros (M€). Therefore, considering the economic feasibility, there are areas where the floating OWF considered for the electric tariff taken into account is economically feasible because the *IRR* is higher than the weighted average cost of capital (WACC) and the *NPV* is greater than zero. On the other hand, *LCOE* (see Figure 5) presents a minimum value of 74.14 €/MWh for the studied region.

**Figure 7:**
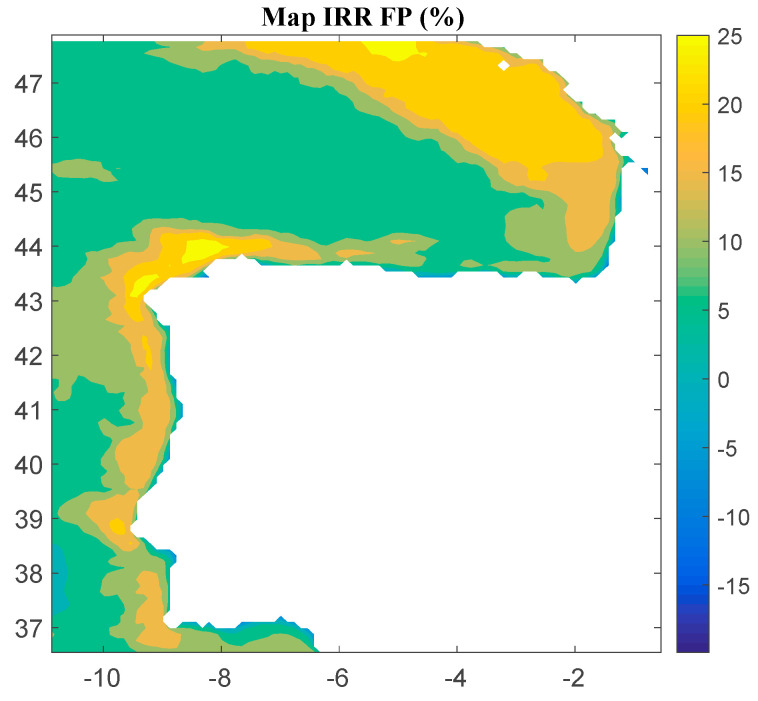
*IRR* (in percentage) for the floating OWF selected using Method 2. Source: Own elaboration.

**Figure 8:**
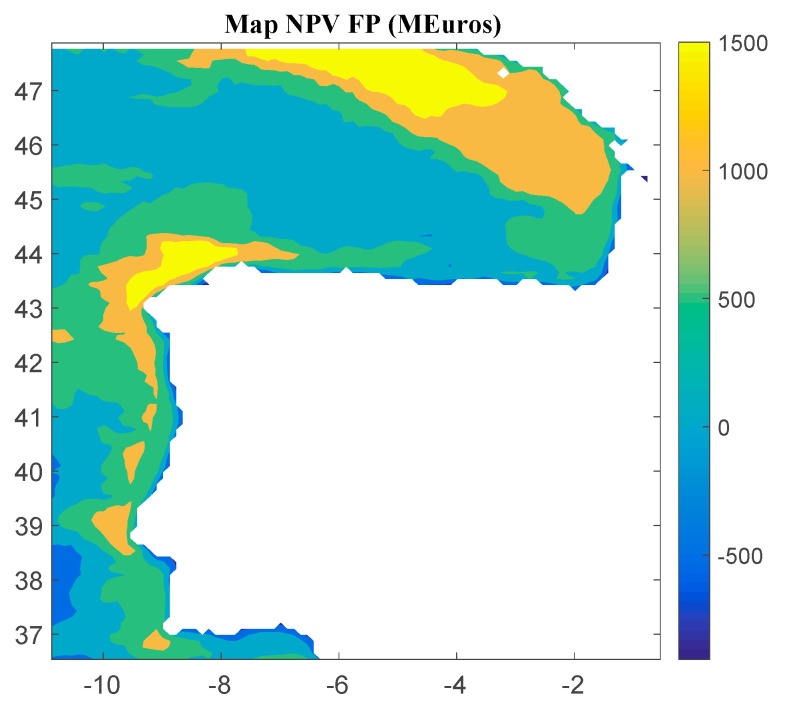
*NPV* (in M€) for the floating OWF selected using Method 2. Source: Own elaboration.

**Figure 9:**
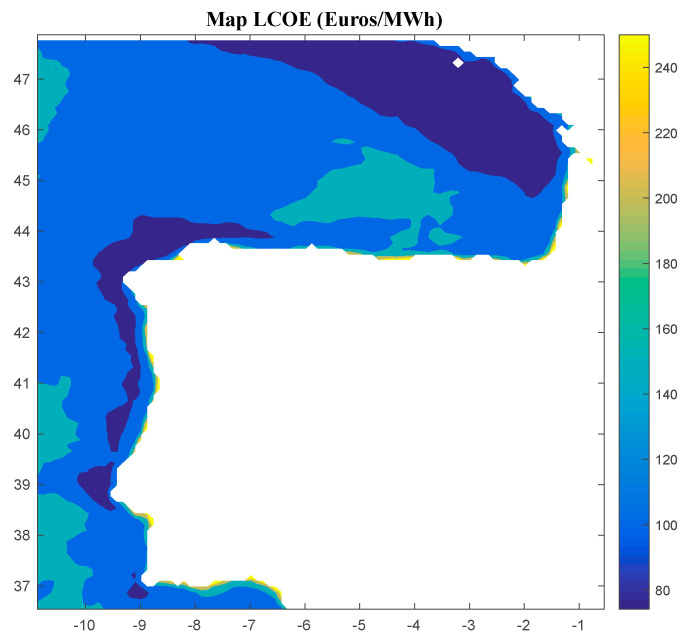
*LCOE* (in €/MWh) for the floating OWF selected using Method 2. Source: Own elaboration.

Considering Method 2, the maps for *IRR*, *NPV*, and *LCOE* are shown in Figure 7, Figure 8 and Figure 9, respectively. The *IRR* varies between −20% and 26%, and *NPV* ranges from −907 to 1739 M€. Consequently, considering the economic feasibility, there are areas where the floating OWF considered for the electric tariff taken into account is economically feasible because the *IRR* is higher than the WACC and the *NPV* is greater than zero. On the other hand, *LCOE* (see Figure 8) presents attractive values for the Galician area (northwest of the Iberian Peninsula), with a minimum value of 74.12 €/MWh.

**Figure 10:**
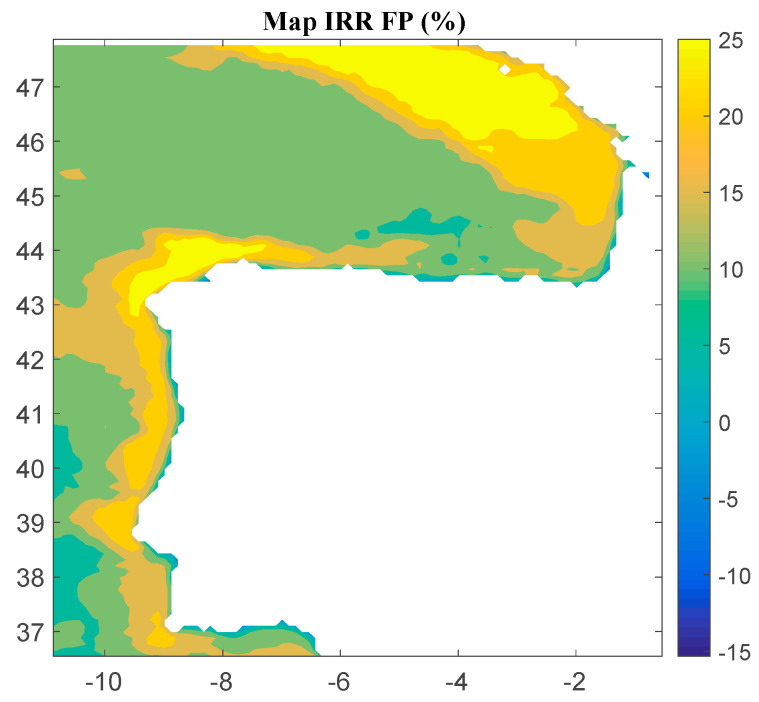
*IRR* (in percentage) for the floating OWF selected using Method 3. Source: Own elaboration.

**Figure 11:**
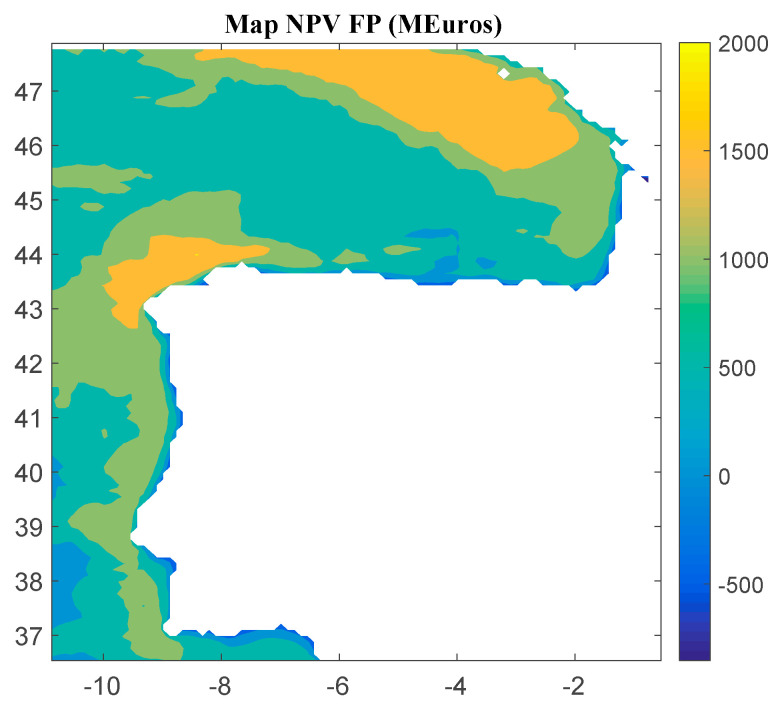
*NPV* (in M€) for the floating OWF selected using Method 3. Source: Own elaboration.

**Figure 12:**
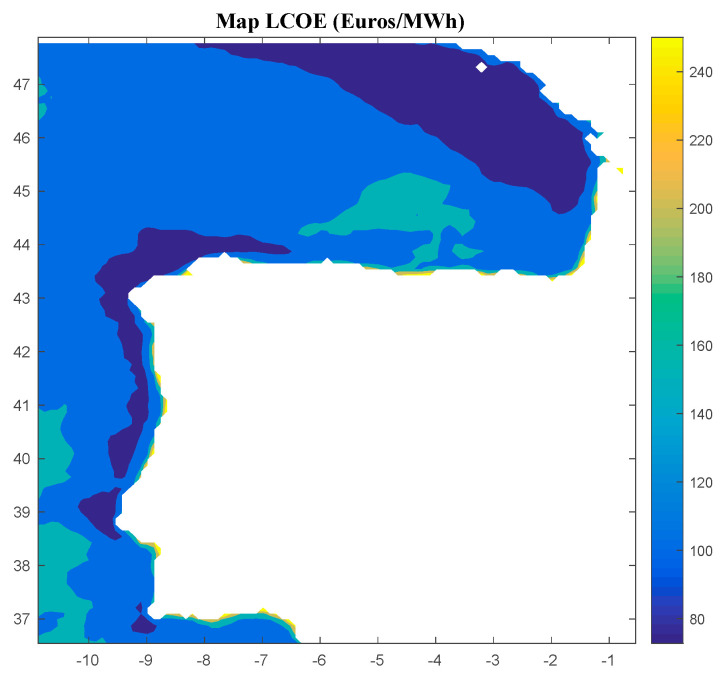
*LCOE* (in €/MWh) for the floating OWF selected using Method 3. Source: Own elaboration.

Considering Method 3, the maps for *IRR*, *NPV*, and *LCOE* are shown in Figure 10, Figure 11 and Figure 12, respectively. The *IRR* ranges from −15% to 29%, and *NPV* ranges from −853 to 2001 M€. Consequently, considering the economic feasibility, there are areas where the floating OWF considered for the electric tariff taken into account is economically feasible because the *IRR* is higher than the WACC and the *NPV* is greater than zero. On the other hand, *LCOE* (see Figure 12) presents attractive values for the Galician area (northwest of the Iberian Peninsula), with a minimum value of 72.78 €/MWh.

**Figure 13:**
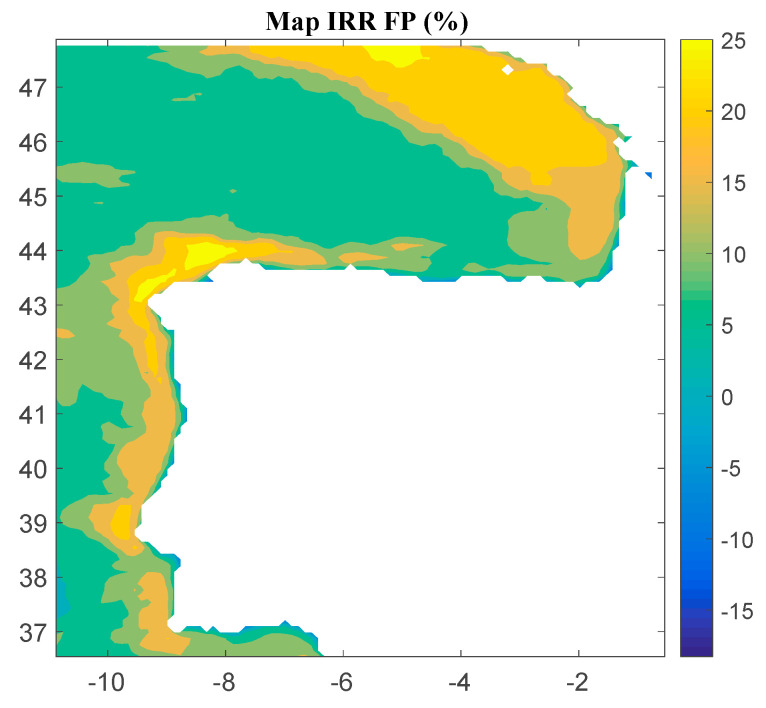
*IRR* (in percentage) for the floating OWF selected using Method 4. Source: Own elaboration.

**Figure 14:**
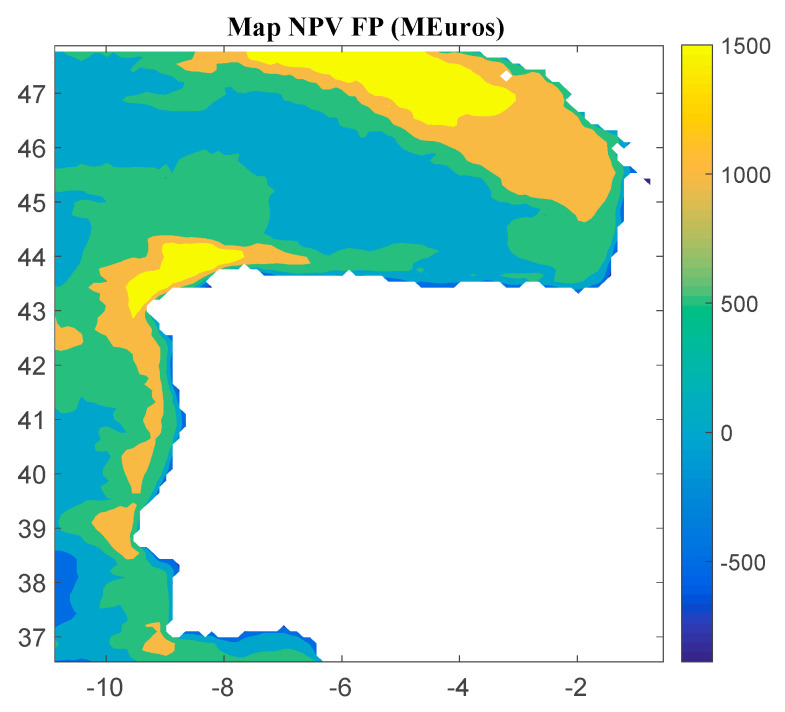
*NPV* (in M€) for the floating OWF selected using Method 4. Source: Own elaboration.

**Figure 15:**
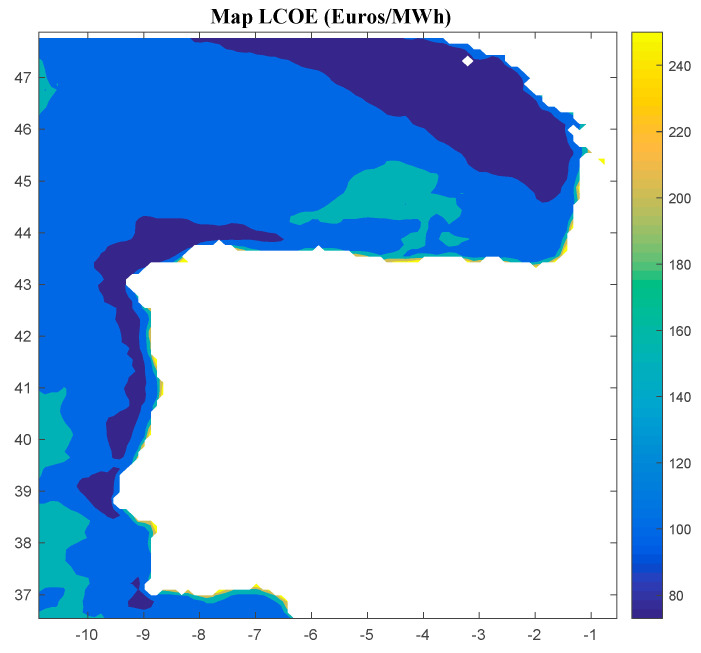
*LCOE* (in €/MWh) for the floating OWF selected using Method 4. Source: Own elaboration.

Considering Method 4, the maps for *IRR*, *NPV*, and *LCOE* are shown in Figure 13, Figure 14 and Figure 15, respectively. The *IRR* ranges from −18% to 27%, and *NPV* ranges from −889 to 1785 M€. Consequently, considering the economic feasibility, there are areas where the floating OWF considered for the electric tariff taken into account is economically feasible because the *IRR* is higher than the WACC and the *NPV* is greater than zero. On the other hand, *LCOE* (see Figure 15) presents attractive values for the Galician area (northwest of the Iberian Peninsula), with a minimum value of 73 €/MWh.

## 4. Discussion

The first step in analyzing the viability of a wind farm at a particular location is to analyze the wind resource at that place. However, considering that the useful life of a wind farm is 20 to 25 years, it is equally important to determine how climate change may influence that wind resource in the near future. Thus, the analysis of future WPD projections under different greenhouse gas emission scenarios is essential. The impacts of climate change on the future potential of European wind power were investigated by developing a mid-century wind power plant scenario to focus the impact assessment on the best locations for the future wind power industry. At the local level, the wind farms in the south of Europe, such as the Iberian and Italian ones, will probably be the most affected. Specifically, it is likely that the energy production of the Iberian Peninsula will be the most affected at the end of the century under the RCP8.5 scenario with a robust projection of a reduction in wind energy of between 5% and 10% on an annual scale, as well as a 15% reduction in autumn. Results obtained in this study are in the range of those of Tobin et al. (2016), who showed a reduction in offshore WPD of between 2% and 6% in practically the entire region, except at Cape Finisterre and between Cape Roca and Cape San Vicente, where no changes are projected for the near future. These changes are considerably smaller than those projected by other authors [18], since changes are relative to the recent past (2001–2020) in the present study, while [18] considered the last decades of the 20th century as the reference period.

Previous studies gave different values for *IRR*, *NPV*, and *LCOE*. As Table 3 shows, the results obtained in this paper are similar to those of other studies.

Table 4 shows the comparison of the best results of the four methods considered, comparing Methods 1, 3, and 4 with Method 2, which is the most suitable theoretically because it represents the real energy of the future years. It is important to note that the most important difference between the results of the four methods is in *NPV*, with a maximum difference of 13.09% for Method 3 with respect to Method 2. However, the value of *LCOE* is very similar in all methods. In fact, the highest difference is 1.84%, which is a very low value in comparison to the percentage of *NPV*.

## 5. Conclusions

Wind energy resources are subject to changes in climate; therefore, the viability and profitability of a wind farm in particular locations should be calculated by considering wind energy density projections in the near future. This study has analyzed the economic feasibility of OWFs along the Atlantic coast, from Brest to Cape St. Vicente, for the next 20 years. For this, past and projected future wind data have been taken into account to analyze their influence on the main economic parameters that determine the feasibility of such wind farms (*NPV*, *IRR*, and *LCOE*). This had never been done before, as previous studies on the profitability of OWFs based their results on the wind resource of recent years.

The main results of this study show that a 2%–6% reduction in WPD is projected for the near future along the northwest coast of the Iberian Peninsula, except for the Cape Finisterre coast and the coastal area between Cape Roca and Cape San Vicente. The future WPD reduction mainly affects the *NPV* of the farm and has little influence on *LCOE*.

Finally, the method proposed in this study provides a good estimate of the economic viability of OWFs since it also takes into account possible wind resource reductions due to climate change over the wind farm lifetime.

## Figures and Tables

**Table 1:** Regional climate simulations from CORDEX (http://www.cordex.org/ (accessed on 20 June 2020) project used in this study. The column labeled GCM indicates the name of the global climate model that forces the regional climate model. The column labeled with RCM indicates the name of the regional climate model, and the last column indicates the name of the institute responsible for the regional climate model.

CORDEX Simulations		
GCM	RCM	INSTITUTE
CNRM-CM5	CCLM4-8-17	CLMcom
CNRM-CM5	RCA4	SMHI
EC-EARTH	RACMO22E	KNMI
IPSL-CM5A-MR	RCA4	SMHI
IPSL-CM5A-MR	WRF331F	INERIS
MPI-ESM-LR	CCLM4-8-17	CLMcom
MPI-ESM-LR	REMO2009	MPI-CSC

**Table 2:** Main characteristics of the farm.

Variable	Value	Unit
Total power of the farm	500	MW
Electric tariff	190	€/MWh
Capital cost	8%	-
Life-cycle of the farm	20	years

**Table 3:** Results of several references.

Reference	Tariff (€/MWh)	Location	*IRR (%)*	*NPV*	*LCOE*
[47]	200	Portugal	−0.37	−193.75 M€	289.49 €/MWh
	200	Portugal	−0.9	−230.08 M€	303.97 €/MWh
	200	Portugal	−2.12	−240.51 M€	325.64 €/MWh
[52]	-	Chile	2–17.3	−713–412 M$	73–1004 $/MWh
		Thailand	10–20	-	188–343 $/MWh

**Table 4:** Comparison of results in percentage relative to Method 2.

Variable	*IRR*	*NPV*	*LCOE*
Method 1	−8.72%	−10.73%	−0.03%
Method 3	−10.24%	−13.09%	1.84%
Method 4	−0.60%	−2.58%	1.53%

## Data Availability

Not applicable
